# Supplementary material for: Psychometric Properties of the Italian Version of the 25-Item Hikikomori Questionnaire for Adolescents
Source: Int J Environ Res Public Health. 2022 Aug 21;19(16):10408. doi: 10.3390/ijerph191610408 (PMC9408307; doi:10.3390/ijerph191610408)
Supplement: Supplementary file 1 [file ijerph-19-10408-s001.zip › ijerph-1798013-supplementary.pdf]

International Journal of Environmental Research and Public Health

Psychometric properties of the Italian version of the 25-item Hikikomori Questionnaire for adolescents

Simone Amendola, Fabio Presaghi, Alan Robert Teo and Rita Cerutti

Table S1. Latent correlations between psychological constructs.

	**1**	**2**	**3**	**4**	**5**
1. Psychoticism	1				
2. Problematic internet use	0.328**	1			
3. Depression	0.703**	0.420*	1		
4. Anxiety	0.543***	0.330**	0.795**	1	
5. PLEs total distress	0.603***	0.358***	0.530**	0.605***	1

PLEs: psychotic-like experiences.
* $p < 0.05$, ** $p < 0.01$, *** $p < 0.001$.
N= 221.

Table S2. Prevalence of psychotic-like experiences (in the last month) according to sex.

	Total sample (N= 221) n (%)	Male (n= 123) n (%)	Female (n= 98) n (%)	Chi square tests of independence $\chi 2(1)$
PLEs total symptoms *(M ± SD)*	7.01 ± 4.19	6.76 ± 4.09	7.33 ± 4.31	(*t*) - 0.991
1. Familiar surroundings seem strange	42 (19)	20 (16.3)	22 (22.5)	1.357
2. Hearing unusual sounds	94 (42.5)	53 (43.1)	41 (41.8)	0.035
3. Things seem different than usual	33 (14.9)	18 (14.6)	15 (15.3)	0.019
4. Paranormal experiences	60 (27.1)	24 (19.5)	36 (36.7)	8.180**
5. Not in control of ideas or thoughts	54 (24.4)	25 (20.3)	29 (29.6)	2.537
6. Difficult getting the point	92 (41.6)	49 (39.8)	43 (43.9)	0.366
7. Being unusually gifted or talented	80 (36.2)	57 (46.3)	23 (23.4)	12.355***
8. People watching you or talking about you	118 (53.4)	60 (48.8)	58 (59.2)	2.372
9. Strange feelings beneath the skin	65 (29.4)	29 (23.6)	36 (36.7)	4.548*
10. Suddenly distracted by distant sounds	73 (33)	41 (33.3)	32 (32.6)	0.011
11. Sense that some person or force is around	56 (25.3)	23 (18.7)	33 (33.7)	6.465*
12. Worry that something is wrong with the mind	60 (27.1)	28 (22.8)	32 (32.6)	2.697
13. Feeling like non-existing, world does not exist	58 (26.2)	33 (26.8)	25 (25.5)	0.049
14. Being confused whether something was real	65 (29.4)	37 (30.1)	28 (28.6)	0.060
15. Beliefs that are unusual or bizarre	102 (46.2)	58 (47.2)	44 (44.9)	0.112
16. Feeling that parts of the body have changed	73 (33)	41 (33.3)	32 (32.7)	0.011
17. Thoughts so strong that can almost hear them	74 (33.5)	37 (30.1)	37 (37.8)	1.442
18. Mistrust or suspicious of other people	137 (62)	71 (57.7)	66 (67.3)	2.144
19. Seeing unusual things like flashes, flames	51 (23.1)	32 (26)	19 (19.4)	1.350
20. Seeing things that other people cannot see	49 (22.2)	36 (29.3)	13 (13.3)	8.095**
21. Hard to understand what is saying	114 (51.6)	60 (48.8)	54 (55.1)	0.873

* $p < 0.05$, ** $p < 0.01$, *** $p < 0.001$.

Figure S1. Distribution of psychotic-like experiences (in the last month) ranked according to the frequency of positive endorsement (response: yes) in the total sample.

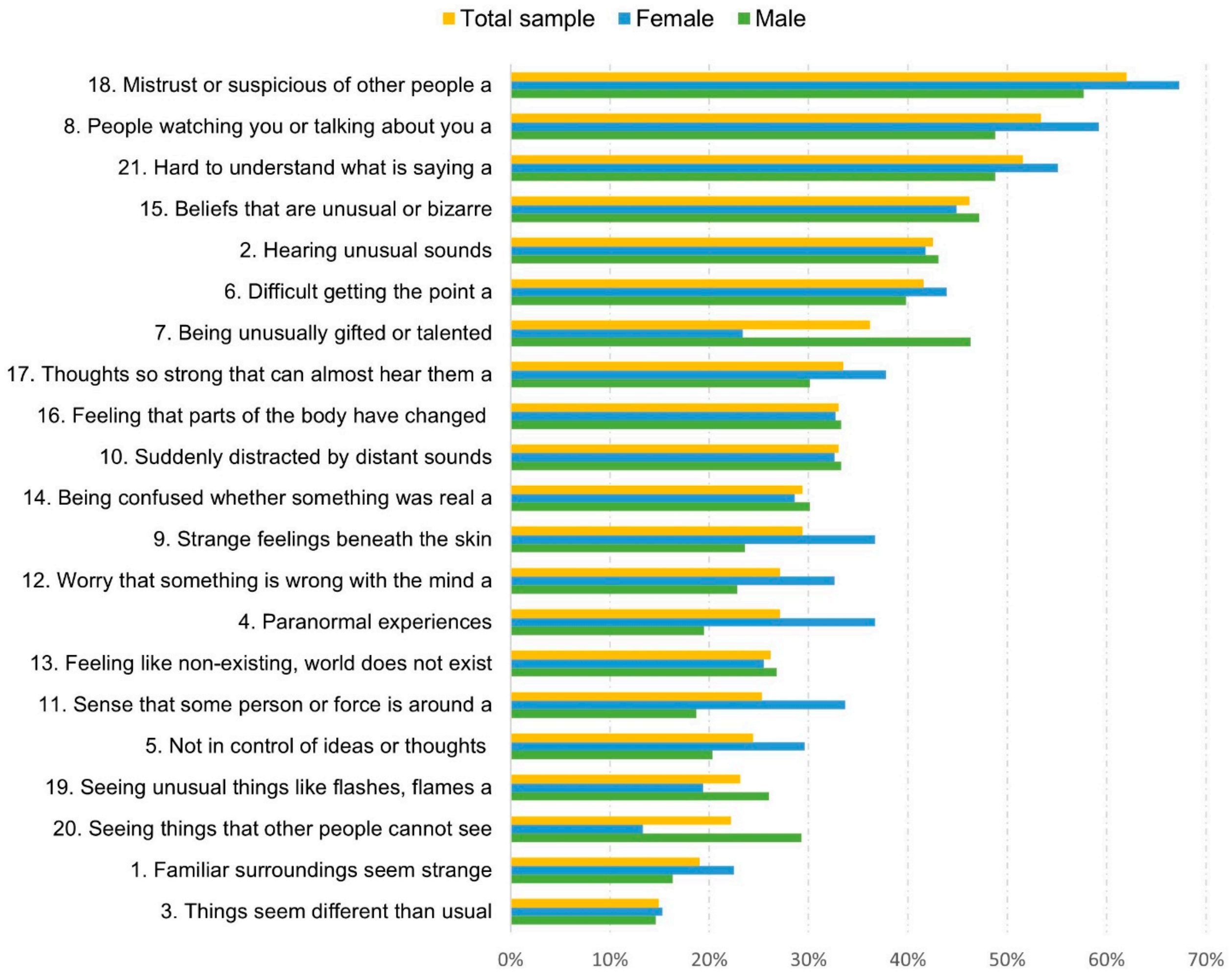

Table S3. Significant differences in prevalence of psychotic-like experiences (in the last month) according to risk for hikikomori (indicated by the HQ-25 cutoff score of 42 or above).

	**Not at-risk (n= 194)** n (%)	**At-risk (n= 27)** n (%)	**Chi square tests of independence $\chi 2(1)$**
PLEs total symptoms *(M ± SD)*	6.69 ± 4.14	9.37 ± 3.82	(*t*) - 3.183**
5. Not in control of ideas or thoughts	43 (22.2)	11 (40.7)	4.429*
8. People watching you or talking about you	98 (50.5)	20 (74.1)	5.286*
12. Worry that something is wrong with the mind	46 (23.7)	14 (51.9)	9.490**
13. Feeling like non-existing, world does not exist	46 (23.7)	12 (44.4)	5.263*
15. Beliefs that are unusual or bizarre	82 (42.3)	20 (74.1)	9.648**
21. Hard to understand what is saying	94 (48.5)	20 (74.1)	6.229*

* $p < 0.05$, ** $p < 0.01$.
